# Effect of *PPAR-γ2* Gene Pro12Ala polymorphism (Rs1801282) and Vitamin D_3_ on glucose homeostasis in Type 2 diabetic subjects from Gujarat-India

**DOI:** 10.1186/1755-8166-7-S1-P37

**Published:** 2014-01-21

**Authors:** Avisek Majumder, Frenny Sheth, Manan Patel, Bhavik Doshi, Navneet Shah, Premal Thankor, Rama Vaidya, Jayesh Sheth

**Affiliations:** 1 FRIGE’s Institute of Human Genetics, FRIGE House, Jodhpur Gam Road, Satellite, Ahmedabad-380015, India; 2 Department of Diabetes and Endocrinology; Sterling Hospital, Ahmedabad- 380052, India; 3 Gujarat Diabetic Association, Ahmedabad-380007, India; 4 Kasturba Health Society, Medical Research Centre, Mumbai-400056, India

## Background

Pro12Ala polymorphism in *PPAR-γ2* gene is known to be involved in insulin sensitization and metabolic deregulation in Type 2 Diabetes (T2D) subject. Considering beneficial effects of Vitamin D_3_ in functional regulation of pancreatic-β cells, present population based study was designed to determine the association of Pro12Ala polymorphism with serum Vitamin D_3_ level and its effects on glucose homeostasis in T2D and non-diabetic subjects.

## Materials & methods

Total 508 subjects (including 210 T2D & 298 controls) were divided into two groups according to serum Vitamin D_3_ level. GrouPI: included 338 subjects (150 T2D out of 338) having Vitamin D_3_ deficiency (≤25.0 nmol/l) and group-II: included 170 subjects (60 T2D out of 170) with normal vitamin D_3_ level (>25.0 nmol/l). All cases were investigated for Vitamin D_3_, glycosylated hemoglobin (HbA1C) level and Pro12Ala variant of *PPAR-γ2* gene.

## Results

It was observed there is 12Ala allele frequency of PPARγ2 gene in 10.19% of T2D and 9.46% in control subjects (p>0.36). The mean HbA1C was better controlled in group-II T2D subjects with 12Ala allele compared to group-I T2D subjects having same allele (7.26±0.44% vs. 8.35±0.43%, p>0.014). In contrary to the above, patients who were homozygous for 12Pro allele, the mean HbA1c remains high irrespective of the normal Vitamin D_3_ levels (8.59±0.18% vs. 8.29±0.28% in group-I and group-II respectively).

## Conclusions

Significant decrease in mean HbA1C level was observed in T2D patients with 12Ala allele and normal Vitamin D_3_ level compared to the patients having same allele with Vitamin D3 deficiency. No such effect was observed in T2D patients with homozygous status for 12Pro allele. This indicate that biologically active form of Vitamin D_3_ (125(OH)D_3_) together with 12Ala allele may affect glucose homeostasis by some gene-nutrition interactions.

